# Prodromal stage of late-onset psychosis

**DOI:** 10.1192/j.eurpsy.2022.464

**Published:** 2022-09-01

**Authors:** V. Pochueva, V. Sheshenin, M. Savina, T. Safarova

**Affiliations:** FSBSI “Mental Health Research Centre”, Geriatric Psychiatry, Moscow, Russian Federation

**Keywords:** Late-onset psychosis, late onset schizophrenia, prodromal, first episod psychosis

## Abstract

**Introduction:**

Prodromal stage of psychosis may be an aim of prevention first episode psychosis. In late-onset psychosis it’s low described.

**Objectives:**

The aim of this investigation is to describe prodromal phase of late-onset psychosis and their connection with psychopathology.

**Methods:**

74 women with late-onset psychosis (age 64,3±6,7) - late-onset schizophrenia (LOS) (n=49, age 63±8,4), schizoaffective disorder (n=17, age 62,4±6,5), late onset delusion disorder (LoDD) (n=8, age 76,6±4,3) underwent clinical assessment (SOPS, PANSS, HAMD-17), cognitive examination (MMSE, MoCA), structured interviewing on life-time pathology. Spearman’s rho statistics was used.

**Results:**

LOP patients have low prodromal symptoms according to the SOPS score. In LOS patients middle score of SOPS is 18±8,5, in schizoaffective patients middle score of SOPS is 12,3±6,8, and for LoDD SOPS is 13,4 ±4,6. Prodromal disturbances are represented mainly by fragmentary paranoid ideas, thoughts of unusual content, acoasms and affective fluctuations. In LOS patients SOPS negative subscale correlate with HAMD (r=0,384, p<0,05), desorganisation subscale correlate with PANSS common psychopathology subscale (r=0,32, p<0,05) and total PANSS (r=0,28, p<0,05), generous subscale and common SOPS correlate with HAMD (r=0,29, r=0,3, p<0,05). For patients with schizoaffective disorder there is no correlations, and in patients with LoDD SOPS desorganisation subscale negative correlate with PANSS negative subscale (r=-0,71, p<0,05).

**Conclusions:**

In patients with late psychoses, the severity of disorders in the prodromal period is minimal. However, prodromal features are associated with the psychopathology.

**Disclosure:**

No significant relationships.

